# Supplementary material for: ORPER: A Workflow for Constrained SSU rRNA Phylogenies
Source: Genes (Basel). 2021 Oct 29;12(11):1741. doi: 10.3390/genes12111741 (PMC8623055; doi:10.3390/genes12111741)
Supplement: Supplementary file 1 [file genes-12-01741-s001.zip › genes-1429420-Supp-files/Supplementary File S1.pdf]

Supplementary File S1

Supplementary Table S1. Information of the 152 ULC strains used in this study, including their strain number, taxonomic name, geographical origin, isolation habitat, recommended growth temperature (°C), recommended growth medium and, if applicable, the corresponding reference.

Strain accronym	Taxonomic name	Geographical origin	Isolation habitat	Recommended growth temperature (°C)	Recommended growth medium	Genbank accession number	Reference
ULC001	*Stenomitos* sp. ANT.L53B.1	Larsemann Hills, East Antarctica, Prydz Bay, Antarctica	Lake 53, microbial mat	18	BG11	AY493608	Taton et al., 2011
ULC004	*Leptolyngbya cf. fragilis* ANT.LH52.1	Larsemann Hills, East Antarctica, Prydz Bay, Antarctica	Lake 52, microbial mat	18	BG11	AY493584	Taton et al., 2011
ULC007	*Phormidesmis priestleyi* ANT.LH52.4	Larsemann Hills, East Antarctica, Prydz Bay, Antarctica	Lake 52, microbial mat	18	BG11	AY493578	Taton et al., 2011
ULC008	*Nostoc* sp. ANT.PROGRESS.2.1	Larsemann Hills, East Antarctica, Prydz Bay, Antarctica	Lake Progress, microbial mat	12	BG110	AY493622	Taton et al., 2012
ULC009	*Plectolyngbya hodgsonii* ANT.PROGRESS2.2[T]	Larsemann Hills, East Antarctica, Prydz Bay, Antarctica	Lake Progress, microbial mat	18	BG11	AY493583	Taton et al., 2011
ULC012	*Plectolyngbya hodgsonii* ANT.GENTNER2.2	Larsemann Hills, East Antarctica, Prydz Bay, Antarctica	Lake Gentner, microbial mat	18	BG11	AY493616	Taton et al., 2011
ULC015	*Nostoc* sp. ANT.LH61.1	Lake 61, Eastern Antarctica, Larsemann Hills, Antarctica	Lake 61, microbial mat	12	BG110	AY493592	Taton et al., 2011
ULC018	*Stenomitos* sp. ANT.LH53B.2	Larsemann Hills, East Antarctica, Prydz Bay, Antarctica	Lake 53, microbial mat	18	BG11	AY493576	Taton et al., 2011
ULC020	*Pegethrix* sp. ANT.MANNING.1	Larsemann Hills, East Antarctica, Prydz Bay, Antarctica	Lake Manning, microbial mat	18	BG11	AY493573	Taton et al., 2011
ULC021	*Phormidesmis priestleyi* ANT.GENTNER2.4	Larsemann Hills, East Antarctica, Prydz Bay, Antarctica	Lake Gentner, microbial mat	18	BG11	AY493580	Taton et al., 2011
ULC022	*Phormidesmis priestleyi* ANT.LH61.2	Larsemann Hills, East Antarctica, Prydz Bay, Antarctica	Lake 61, microbial mat	18	BG11	AY493582	Taton et al., 2011
ULC023	*Leptolyngbya cf. antarctica* ANT.FIRELIGHT.1	Larsemann Hills, East Antarctica, Prydz Bay, Antarctica	Lake Firelight, microbial mat	12	BG11	AY493590	Taton et al., 2011
ULC024	*Leptolyngbya* cf. *fragilis* ANT.RAUER8.1	Rauer Island, East Antarctica, Prydz Bay, Antarctica	Lake Rauer 8, microbial mat	18	BG11	AY493619	Taton et al., 2011
ULC025	*Pegethrix* sp. ANT.LH70.1	Larsemann Hills, East Antarctica, Prydz Bay, Antarctica	Lake 70, microbial mat	18	BG11	AY493574	Taton et al., 2011
ULC027	*Leptolyngbya antarctica* ANT.PROGRESS2.5	Larsemann Hills, East Antarctica, Prydz Bay, Antarctica	Lake Progress, microbial mat	18	BG11	AY493620	Taton et al., 2011
ULC029	*Stenomitos* sp. ANT.LH52B.3	Larsemann Hills, East Antarctica, Prydz Bay, Antarctica	Lake 52, microbial mat	12	BG11	AY493612	Taton et al., 2011
ULC030	*Plectolyngbya hodgsonii* ANT.LH52B.4	Larsemann Hills, East Antarctica, Prydz Bay, Antarctica	Lake 52, microbial mat	18	BG11	AY493616	Taton et al., 2011

ULC031	*Shackletoniella antarctica* ANT.LH18.1(T)	Larsemann Hills, East Antarctica, Prydz Bay, Antarctica	Lake 18, microbial mat	18	BG11	AY493607	Taton et al., 2011
ULC032	*Shackletoniella antarctica* ANT.GENTNER2.5	Larsemann Hills, East Antarctica, Prydz Bay, Antarctica	Lake Gentner, microbial mat	18	BG11	AY493603	Taton et al., 2011
ULC034	*Wilmottia murrayi* ANT.PENDANT.1	Vestfold Hills, East Antarctica, Prydz Bay, Antarctica	Lake Pendant, microbial mat	18	BG11	AY493626	Taton et al., 2011
ULC035	*Phormidium priestleyi* ANT.ACEV5.1	Vestfold Hills, East Antarctica, Prydz Bay, Antarctica	Ace Lake, microbial mat	12	BG11	AY493586	Taton et al., 2011
ULC036	*Shackletoniella antarctica* ANT.WATTS.1	Vestfold Hills, East Antarctica, Prydz Bay, Antarctica	Lake Watts, microbial mat	18	BG11	AY493604	Taton et al., 2011
ULC037	*Shackletoniella antarctica* ANT.LH18.2^T	Larsemann Hills, East Antarctica, Prydz Bay, Antarctica	Lake 18, microbial mat	12	BG11	AY493606	Taton et al., 2011; Strunecky et al., 2020
ULC038	*Nostoc* sp. ANT.LH52B.5	Larsemann Hills, East Antarctica, Prydz Bay, Antarctica	Lake 52, microbial mat	18	BG110	AY493596	Taton et al., 2011
ULC041	*Leptolyngbya* cf. *antarctica* ANT.ACE.1	Vestfold Hills, East Antarctica, Prydz Bay, Antarctica	Ace Lake, microbial mat	18	BG11	AY493588	Taton et al., 2011
ULC046	*Nostoc* sp. ANT.GENTNER2.6	Larsemann Hills, Broknes Peninsula, East Antarctica, Prydz Bay, Antarctica	Lake Gentner 2, microbial mat	12	BG110	AY493595	Taton et al., 2011
ULC047	*Leptolyngbya* cf. *antarctica* ANT.ACEV6.1	Vestfold Hills, East Antarctica, Prydz Bay, Antarctica	Ace Lake, microbial mat	18	BG11	AY493589	Taton et al., 2011
ULC049	*Phormidesmis priestleyi* ANT.LH66.1	Larsemann Hills, East Antarctica, Prydz Bay, Antarctica	Lake 66, microbial mat	18	BG11	AY493581	Taton et al., 2011
ULC057	*Stenomitos* ANT.REIDJ.1	Larsemann Hills, East Antarctica, Prydz Bay, Antarctica	Lake Reid, microbial mat	18	BG11	AY493611	Taton et al., 2011
ULC060	*Scytonema* sp. ANT.LG2.8	Larsemann Hills, East Antarctica, Prydz Bay, Antarctica	Lake Gentner, microbial mat	12	BG11	AY493624	Taton et al., 2011
ULC065	*Cyanobium* sp. O-154	Bylot Island, Arctic, Canada	Unknown	12	BG11	MH118733	
ULC066	*Pseudanabaena frigida* O-155	Bylot Island, Arctic, Canada	Unknown	12	BG11	MK139957	
ULC069	*Pseudanabaena frigida* O-302	Québec, Québec, Sub-arctic, Canada	Lake with clear water	12	BG11	MH118736	
ULC070	*Pseudanabaena frigida* O-401	Québec, Québec, Sub-arctic, Canada	Lake with clear water	12	BG11	MH118737	
ULC073	*Leptolyngbya glacialis* TM1FOS73	Dufek Massif, Transantarctic Mountains, Antarctica	Bottom brine of Forlidas Pond	12	BG11	EU852495	Fernández-Carazo et al. 2011
ULC074	*Phormidium priestleyi* O-067	Mc Murdo Ice Shelf, South Victoria Land, Antarctica	Unknown	12	BG11	MH118739	
ULC076	*Phormidium autumnale* O-151	Bylot Island, Arctic, Canada	Unknown	12	BG11	MH118740	
ULC080	*Anabaena* sp. CY-036	Unknown, New Zealand	Unknown	12	BG110	MH118743	
ULC081	*Cyanobium* sp. Limnopolar	Limnopolar Lake, South Shetland islands, Livingston Island, Antarctica	Unknown	12	BG11	MK139958	
ULC082	*Cyanobium* sp.Chester Cone	Chester Cone, South Shetland islands, Livingston Island, Antarctica	Unknown	12	BG11	MH118744	
ULC084	*Cyanobium* sp. Laguna Chica	Laguna Chica, South Shetland islands, Livingston Island, Antarctica	Unknown	12	BG11	MK139959	
ULC088	*Anabaena* cf. *oscillarioides* S88	Unknown	Unknown	12	BG110	OK576186	

ULC090	*Leptolyngbya* cf. *antarctica* ANT-SOS	Mc Murdo Ice Shelf, South Victoria Land, Bratina Island, Antarctica	Son of salt (SOS) pond	12	BG11	AY493588	Nadeau et al., 2001
ULC092	*Phormidium pseudopriestleyi* ANT - SHADE # 7	Mc Murdo Ice Shelf, South Victoria Land, Bratina Island, Antarctica	Shading pond	12	BG11	OK576187	Nadeau et al., 2001
ULC093	*Phormidium pseudopriestleyi* ANT-SALT	Mc Murdo Ice Shelf, South Victoria Land, Bratina Island, Antarctica	Salt pond	12	BG11	AF263337	Nadeau et al., 2001
ULC095	*Phormidium autumnale* ANT-PINNACLE#4	Mc Murdo Ice Shelf, South Victoria Land, Bratina Island, Antarctica	Pinnacle pond	12	BG11	MK139960	Nadeau et al., 2001
ULC097	*Phormidium autumnale* ANT-LUNCH	Mc Murdo Ice Shelf, South Victoria Land, Bratina island, Antarctica	Pond lunch	12	BG11	AF263335	Nadeau et al., 2001
ULC100	*Geitlerinema* sp. ANT-CASTEN #2	Mc Murdo Ice Shelf, South Victoria Land, Bratina Island, Antarctica	Unknown	12	BG11	OK576188	Nadeau et al., 2001
ULC102	*Phormidium pseudopriestleyi* ANT-BRACK -2	Mc Murdo Ice Shelf, South Victoria Land, Bratina Island, Antarctica	Brack Pond	12	BG11	AF263331	Nadeau et al., 2001
ULC104	*Shackletoniella* sp. ANT-BLACK - 2	Mc Murdo Ice Shelf, South Victoria Land, Bratina Island, Antarctica	Black pond	12	BG11	AF263331	Nadeau et al., 2001
ULC106	*Phormidium pseudopriestley*ANT-G17_OSC	Mc Murdo Ice shelf, South Victoria Land, Bratina Island, Antarctica	Saline pond	12	BG11	OK576189	Nadeau et al., 2001
ULC107	*Microcoleus favosus* JR1	Komarek's seepages, Antarctic Peninsula, James Ross Island, Antarctica	Brown mats in spring area, Slope Creek	12	BG11	JN230327	Strunecký et al. 2013
ULC110	*Microcoleus* sp. JR5	Komarek's seepages, Antarctic Peninsula, James Ross Island, Antarctica	Black biofilm on rocks	18	BG11	JN230334	Strunecký et al. 2013
ULC112	*Phormidium priestleyi* JR7	Green Lake, Antarctic Peninsula, James Ross Island, Antarctica	Periphyton in littoral of Green Lake, dark-green	18	BG11	JN230328	Strunecký et al. 2011
ULC115	*Leptolyngbya* sp. JR12	Unknown, Antarctic Peninsula, James Ross Island, Antarctica	Unknown	18	BG11	JN230335	Strunecký et al. 2012
ULC120	*Microcoleus favosus* JR20	Antarctic Peninsula, James Ross Island, Antarctica	Rocks wetted by seep	18	BG11	JN230332	Strunecký et al. 2013
ULC121	*Geitlerinema* sp. JR21	Antarctic Peninsula, James Ross Island, Antarctica	Unknown	12	BG11	KT315945	
ULC127	*Phormidium priestley*i JR27	Antarctic Peninsula, James Ross Island, Antarctica	Unknown	12	BG11	OK576190	
ULC130	*Phormidium murrayi* TM2FOS130	Forlidas pond, Transantarctic Mountains, Dufek massif, Antarctica	Microbial mat of the littoral zone	12	BG11	EU852498	Fernández-Carazo et al. 2011
ULC137	*Sodalinema komarekii* Tsagan-Nur	Tsagan-Nur lake, Siberia, Russian Federation	Biofilm on the littoral of the lake	20-25	Zarrouk+	OK576191	
ULC138	*Sodalinema komarekii* Borzinskoye	Siberia, Russian Federation	Lake Borzinskoye	20-25	Zarrouk+	OK576192	
ULC139	*Sodalinema komarekii* Khil 10-07	Transbaikalian area, Buryat Republic, Siberia, Russian Federation	Lake Khilganta	18	Zarrouk	OK576193	
ULC144	*Leptolyngbya* cf. *antarctica* Kir D8	Southern pa20-25 of Buryat Republic, Siberia, Buryat Republic, Russian Federation	Lake Kiranskoye	20-25	Zarrouk	OK576194	
ULC146	*Nostoc* sp. ANT.UTS. 183	Utsteinen ridge (parcel 21), Dronning Maud land, Sør Rondane Mountains, Antarctica	Black microbial mats and gravel	18	BG110	HM101226	Fernández-Carazo et al. 2012
ULC147	*Phormidesmis priestleyi* ANT.UTS.195	Utsteinen Nunatak, Dronning Maud Land, Sør Rondane Mountains, Antarctica	Black microbial mats on gravel near snow Western side of the Utsteinen Nunatak	12	BG11	HM101225	Fernández-Carazo et al. 2012

ULC149	*Hassallia andreassenii* AW20	Second nunatak of Pingvinane range, Dronning Maud Land, Sør Rondane Mountains, Antarctica	Small stones between bigger stones	12	BG110	OK576195	
ULC153	*Hassallia andreassenii* PCR8	Dronning Maud Land, Sør Rondane Mountains, Antarctica	Unknown	18	BG110	OK576196	
ULC174	*Phormidium lumbricale* OTC8	Tanngarden, Dronning Maud Land, Sør Rondane Mountains, Antarctica	Granite outcrop on the northern side of Tanngarden, black biofilms on granitic gravel	12	BG11	OK576197	
ULC180	*Nostoc* sp. OTC7	Tanngarden, Dronning Maud Land, Sør Rondane Mountains, Antarctica	Granite outcrop on the northern side of Tanngarden, black biofilms on granitic gravel	12	BG110	OK576198	
ULC186	*Leptolyngbya* sp. FW074	Lasne, Wallonia, Belgium	Lake Renipont water sample	20-25	BG11	MK280772	
ULC188	*Cyanobium* sp. FW064	Silenrieux (Eau d'Heure), Wallonia, Belgium	Lake Féronval, water sample	20-25	BG11	OK576199	
ULC191	*Cyanobium* sp. KOTOKEL 10,1	Unknown, Buryat Republic, Russian Federation	Freshwater from lake Kotokel	20-25	BG11	OK576200	
ULC194	*Chroococcidiopsis* sp. PB1101	Perlebandet, Dronning Maud Land, Sør Rondane Mountains, Antarctica	Weathered marble outcrop, under patch of snow	12	BG11	OK576201	
ULC195	*Chroococcidiopsis* sp. SV1103	Svindlandfjellet, Dronning Maud Land, Sør Rondane Mountains, Antarctica	Big boulder, endolith on the upper North surface in cracks with very small white and black lichens	12	BG11	OK576202	
ULC197	*Chroococcidiopsis* sp. PB1113	Perlebandet, Dronning Maud Land, Sør Rondane Mountains, Antarctica	On the scree of garnet-biotite gneiss. In the depression of the rock, near the snow. Lichen crust wet	12	BG11	OK576203	
ULC307	*Phormidium autumnale* P4	First nunatak of the Pingvinane system, westerly from Utsteinen, Dronning Maud Land, Sør Rondane Mountains, Antarctica	Sample of Prasiola in a locality with a skua nest and a lot of remains of Snow Petrels	20-25	BG11	OK576204	
ULC345	*Leptolyngbya* sp. P.RUS1	Moorea, French Polynesia, France	Tissue of coral Porites rus	20-25	ASNIII	OK576205	
ULC346	*Myxosarcina* sp. P.RUS2	Moorea, French Polynesia, France	Tissue of coral Porites rus	20-25	ASNIII	OK576206	
ULC367	*Pegethrix frigida* KOVACIK ANT 2003/73	Keller Peninsula, King Georges Island, Admiralty Bay, Antarctica	Dry epilithic crust in seepage	12	BG11	JN979965	Jancusova et al. 2016
ULC369	*Wilmottia murrayi* KOVACIK ANT 2003/15	Keller Peninsula, King Georges Island, Admiralty Bay, Antarctica	Black skinny crust on moss near the Catharacta maccormicki nest	12	BG11	JN979964	Jancusova et al. 2016
ULC371	*Microcoleus attenuatus* KOVACIK ANT 2003/43	Keller Peninsula, King Georges Island, Admiralty Bay, Antarctica	Dark green growth on remainder of whale bone near seashore	12	BG11	JN979958	Jancusova et al. 2016
ULC373	*Microcoleus favosus* KOVACIK ANT 2004/50	Copacabana, King Georges Island, Admiralty Bay, Antarctica	Green soil powder near a penguin rookery side	12	BG11	JN979959	Jancusova et al. 2016
ULC376	*Wilmottia murrayi* KOVACIK ANT 2004/75	Whalers Bay, Antarctic Peninsula, Deception Island, Antarctica	Mud from a littoral shallow coastal pool	12	BG11	JN979963	Jancusova et al. 2016

ULC381	*Geitlerinema* sp. L2	Goma, Congo	Lac Vert, water sample	20-25	BG11	OK586761	
ULC384	*Leptolyngbya* sp. L6	Goma, Congo	Lac Vert, water sample	20-25	BG11	OK586762	
ULC389	*Pseudanabaena* sp. L27	Goma, Congo	Lac Vert, water sample	20-25	BG11	OK586763	
ULC397	*Nostoc* sp. TG3	Moraine slope west of the Utsteinen nunatak, Dronning Maud Land, Sør Rondane Mountains, Antarctica	Small gravel particles between the rocks, moraine	12	BG110	OK586764	
ULC401	*Timaviella circinata* GR4^{T}	Trieste, Italy	Lampenflora, on rock surface in the Giant Cave	18	BG11	LT634149	Sciuto et al. 2017
ULC402	*Timaviella karstica* GR13^{T}	Trieste, Italy	Lamenflora growing on rock surface in the Giant Cave	18	BG11	LT634150	Sciuto et al. 2017
ULC403	*Phormidium autumnale* FB2	Hverfell volcano, Sub-arctic, Iceland	Fine particles of rhyolite dust	20-25	BG11	OK586765	
ULC406	*Tychonema bornetii* YLC011	Cape Day, South Victoria Land, Antarctica	Microbial mat	18	BG11	OK586766	
ULC409	*Nostoc* sp. TG10	Utsteinen Ridge, Dronning Maud Land, Sør Rondane Mountains, Antarctica	Soil with dark green fragments	18	BG110	OK586767	
ULC415	*Geitlerinema* sp. SHC	Gilan Province, Caspian Sea, Iran	Unknown	18	BG11	OK586768	
ULC417	*Geitlerinema amphibium* Gor-1	Kurdistan, Iran	BaBa Gorgor mineral water spring	18	BG11	OK586769	
ULC421	*Nodularia spumigena* CCY9414	Bornholm Sea, Germany	Surface water	18	2/3 BG110 + 1/3 ASNI	CP007203	Kopf et al. 2015
ULC422	*Thermoleptolyngbya albertanoae* ETS-08	Padova, Euganean Thermal District, Italy	Montegrotto Terme	20-25	BG11	FM210757	Sciuto et al. 2016
ULC424	*Leptolyngbya ectocarpi* C86	STARESO station, Corsica, Calvi, France	Epiphytic on Halopteris scoparia growing on a quay	25	ASNIII	OK586770	Ohki et al. 2004; Wilmotte et al., 1988
ULC426	*Phormidium ambiguum* HK2	Lake Winnipeg, Manitoba, Canada	Boat harbor beach, splash zone at the beach area	12	BG11	OK586771	
ULC428	*Cyanobium* sp. Torca	Laguna Torca National Reserve, Maule Region, Chile	Lake Torca	20-25	BG11	OK586772	
ULC429	*Leptolyngbya gracilis* POS1	Bay of the scientific station STARESO (La Revellata), Corsica, Calvi, France	Epiphytic on a rhizome of Posidonia oceanica	18	ASNIII	OK586773	
ULC431	*Leptolyngbya* sp. LK1	Lake Katinda, Uganda	Lake water	20-25	BG11	OK586774	
ULC437	*Jaaginema subtilissimum* La Gombe 3	Quarry of La Gombe, Esneux, Belgium	Elodea growing on rocks, 4 to 5 m depth underwater	20-25	BG11	OK586775	
ULC441	*Phormidium papyraceum* BOTA3	Botany Institute B22, Sart-Tilman campus, Belgium	Bloc of concrete	18	BG11	OK586776	
ULC444	*Arthrospira platensis* Lonar	Lonar Lake, India	Alkaline Lake	20-25	Zarrouk	OK586777	
ULC445	*Arthrospira platensis* Paracas	Unknown	Unknown	20-25	Zarrouk	OK586778	
ULC447	*Nostoc* sp. TG11	Utsteinen Ridge, Dronning Maud Land, Sør Rondane Mountains, Antarctica	Brown soil under lichen on the Utsteinen Ridge	18	BG110	OK586779	
ULC448	*Spirulina subsalsa* BLCC-M33	Black Point Marina, Florida, USA	Benthic	20-25	ASNIII	OK576182	
ULC449	*Leptolyngbya* sp. BLCC-M10	Everglades National Park, Florida, USA	Benthic	20-25	ASNIII	OK576183	

ULC453	*Leptolyngbya* sp. BLCC-M28	Jupiter Park, Florida, USA	Benthic	20-25	ASNIII	OK576184	
ULC454	*Vermifilum ionodolium* BLCC-M29^{T}	Black Point Marina, Florida, USA	Benthic	20-25	ASNIII	MT321587	Berthold et al., 2021b
ULC457	*Vermifilum ionodolium* BLCC-M32	Black Point Marina, Florida, USA	Benthic	20-25	ASNIII	MT321584	Berthold et al., 2021b
ULC468	*Cyanobium* sp. BS5-0	Bornholm Sea, Baltic sea, Danemark	10 m depth water	18	2/3 BG11 + 1/3 ASNII	AF330253	Ernst et al., 2003
ULC471	*Cyanobium* sp. BS4-0	Bornholm Sea, Baltic sea, Danemark	10 m depth water	18	2/3 BG11 + 1/3 ASNII	AF330252	Ernst et al., 2003
ULC483	*Cyanobium* sp. 6007	Latyan dam, Iran	Surface water samples (50 cm depth)	20-25	BG11	OK586780	Tavakoli et al., 2021
ULC484	*Leptolyngbya* sp. 6008	Taleqan dam, Iran	Surface water samples (50 cm depth)	20-25	BG11	OK575975	
ULC500	*Geitlerinema nematodes* W1.1.1	Vlaams Brabant, Tervuren, Belgium	Ornemental pond	20-25	BG11	OK586781	
ULC503	*Geitlerinema* cf. *ionicum* W1.2.2	Vlaams Brabant, Tervuren, Belgium	Ornemental pond	20-25	BG11	OK586782	
ULC504	*Geitlerinema* cf. *ionicum* W2.1.2	Vlaams Brabant, Tervuren, Belgium	Ornemental pond	20-25	BG11	OK586783	
ULC508	*Pseudoanabaena persicina* POS	Bay of the scientific station STARESO (La Revellata), Corsica, Calvi, France	Epiphytic on a rhizome of Posidonia oceanica	18	ASNIII	OK586784	
ULC522	*Neolyngbya arenicola* BLCC-M52	Indian River Lagoon, Florida, USA	Benthic	20-25	BG11 marine	MT371813	Lefler et al. 2021
ULC525	*Affixifilum floridanum* BLCC-M61^{T}	Elliott Key, Florida, USA	Benthic	20-25	BG11 marine	MT371827	Lefler et al. 2021
ULC529	*Vermifilum ionodolium* BLCC-M65	Biscayne National Park, Florida, USA	Benthic	20-25	BG11 marine	MT321592	Berthold et al., 2021b
ULC530	*Neolyngbya biscaynensis* BLCC-M69^{T}	Biscayne National Park, Florida, USA	Benthic	20-25	BG11 marine	MT371816	Lefler et al. 2021
ULC535	*Neolyngbya biscaynensis* BLCC-M95	Biscayne National Park, Florida, USA	Benthic	20-25	BG11 marine	MT371819	Lefler et al. 2021
ULC542	*Affixifilum granulosum* BLCC-M111	Cannon Beach, John Pennekamp Coral Reef State Park, Florida, USA	Benthic	20-25	BG11 marine	MT371834	Lefler et al. 2021
ULC544	*Brasilonema santannae* BLCC-T43^{T}	Mid Florida Research and Education Center, Florida, USA	Aerophytic, terrestrial	20-25	BG11 marine	MT396506	Barbosa et al. 2021
ULC545	*Brasilonema tolantongensis* BLCC-T61	Mid Florida Research and Education Center, Florida, USA	Aerophytic, terrestrial	20-25	BG11 marine	MT396509	Barbosa et al. 2021
ULC546	*Brasilonema santannae* BLCC-T64	Mid Florida Research and Education Center, Florida, USA	Aerophytic, terrestrial	20-25	BG11 marine	MT396507	Barbosa et al. 2021
ULC547	*Brasilonema octagenarum* BLCC-T71	Mid Florida Research and Education Center, Florida, USA	Aerophytic, terrestrial	20-25	BG110	MT396511	Barbosa et al. 2021
ULC548	*Brasilonema fioreae* BLCC-T72^{T}	Mid Florida Research and Education Center, Florida, USA	Aerophytic, terrestrial	20-25	BG110	MT396512	Barbosa et al. 2021
ULC550	*Brasilonema octagenarum* BLCC-T74	Mid Florida Research and Education Center, Florida, USA	Aerophytic, terrestrial	20-25	BG110	MT396515	Barbosa et al. 2021
ULC551	*Brasilonema fioreae* BLCC-T83	Mid Florida Research and Education Center, Florida, USA	Aerophytic, terrestrial	20-25	BG110	MT396516	Barbosa et al. 2021

ULC557	*Neolyngbya arenicola* BLCC-M60	Biscayne National Park, Florida, USA	Benthic	20-25	BG11 marine	MT371815	Lefler et al. 2021
ULC559	*Spirulina subsalsa* BLCC-M70	Biscayne National Park, Florida, USA	Benthic	20-25	BG11 marine	OK576185	
ULC567	*Limnoraphis* BLCC-M92	Guerande, Salt Marshes, France	Benthic	20-25	BG11 marine	MZ127478	
ULC573	*Brasilonema wernerae* BLCC-T49[T]	Mid Florida Research and Education Center, Florida, USA	Aerophytic, terrestrial	20-25	BG110	MT396508	Barbosa et al. 2021
ULC574	*Brasilonema tolantongensis* BLCC-T51	Mid Florida Research and Education Center, Florida, USA	Aerophytic, terrestrial	20-25	BG110	MT396510	Barbosa et al. 2021
ULC575	*Iningainema tapete* BLCC-T55[T]	Mid Florida Research and Education Center, Florida, USA	Aerophytic, terrestrial	20-25	BG110	MT672265	Berthold et al., 2021a
ULC586	*Neolyngbya regalis* BLCC-M54	Hollywood Beach, Florida, USA	Benthic	20-25	BG11 marine	MT371824	Lefler et al. 2021
ULC588	*Affixifilum granulosum* BLCC-M117	Cannon Beach, John Pennekamp Coral Reef State Park, Florida, USA	Benthic	20-25	BG11 marine	MT371829	Lefler et al. 2021
ULC590	*Johannesbaptistia floridana* BLCC-M67[T]	Biscayne National Park, Florida, USA	Benthic mat on coastal marine sediment	20-25	BG11 marine	MT321576	Berthold et al., 2020
ULC591	*Parakomarekiella sesnandensis* COI00088998[T]	Coimbra, Portugal	Biodeteriorated walls of the Old Cathedral of Coimbra	20-25	BG110	MT044191	Soares et al., 2020
ULC597	*Leptochromothrix valpauliae* BLCC-M82[T]	Biscayne National Park, Florida, USA	Benthic	20-25	BG11 marine	MT321579	Berthold et al., 2021b
ULC598	*Leptochromothrix engenei* BLCC-M83	Biscayne National Park, Florida, USA	Benthic	20-25	BG11 marine	MT321578	Berthold et al., 2021b
ULC599	*Ophiophycus aerugineus* BLCC-M88[T]	Biscayne National Park, Florida, USA	Benthic	20-25	BG11 marine	MT321581	Berthold et al., 2021b
ULC600	*Ophiophycus aerugineus* BLCC-M93	Biscayne National Park, Florida, USA	Benthic	20-25	BG11 marine	MT321582	Berthold et al., 2021b
ULC601	*Ophiophycus aerugineus* BLCC-M103	Twin Rivers Park, Port Salerno Waterfront District, Staurt. Florida, USA	Benthic	20-25	BG11 marine	MT321580	Berthold et al., 2021b
ULC602	*Aphanizomenon* sp. BL5	Unknown, Vlaams Brabant, Brussels Hoofdstedelijk gewest, Belgium	Lake	20-25	50% BG110	OK586785	
ULC604	*Chroococcus* sp. waterbottle	Neupré, Belgium	Static tab water	20-25	BG11	OK586786	
ULC610	*Planktothrix* sp. aqua2	Neupré, Belgium	Aquarium	20-25	BG11	OK586787	
ULC611	*Dolichospermum* sp. BL7	Rue de pecheries,Watermaal-Bosvoorde, Brussels Hoofdstedelijk Gewest, Belgium	Pond	20-25	BG110	OK586788	
ULC718	*Cephalothrix komarekiana* sp. Nov[T]	Pantanal da Nhecolandia, Mato Grosso do sul, Brazil	Alkaline Lake	20-25	BG11	KJ994514	da Silva Malone et al., 2017

Description of culture medium

The growth medium description for BG11, BG110 and ASNII can be found in Rippka et al. (1979), and for Zarrouk in Zarrouk (1966). "50% BG110" is BG110 medium diluted twice in MilliQ water; "BG11 marine" is composed of BG11 with an addition of 35 g/L of aquarium sea salts; "2/3 BG110 + 1/3 ASNI" contains two third of BG110 and one third of ASNIII medium; "Zarrouk+", is prepared as Zarrouk medium, but with an addition of 197 g/l of Na_2CO_3.

SSU rRNA sequences

To test ORPER, we used 152 SSU (16S) rRNA sequences from the cyanobacteria strains present in the BCCM/ULC Cyanobacteria collection (https://bccm.belspo.be/about-us/bccm-ulc). BCCM/ULC is a public collection that aims to gather a representative portion of terrestrial, freshwater and marine cyanobacterial strains with a focus on the polar biodiversity. The collection is part of the BCCM consortium (Belgian Coordinated Collections of Microorganisms).

SSU rRNA sequences from publicly available strains were obtained from GenBank (accession numbers are listed in the Supplementary Table 1) and new SSU rRNA sequences were obtained and deposited in GenBank (Supplementary Table 1). To generate the new SSU rRNA sequences, genomic DNA was extracted from the cyanobacteria using the NucleoSpin Soil kit (Macherey-Nagel, Germany) according the suppliers protocol. The SSU rRNA-ITS region was amplified using the 359F (5' GGGGAATTTTCCGCAATGGG 3') (Nübel et al., 1997) and the 23S30R (5' CTTCGCCTCTGTGTGCCTAGGT 3') (Taton et al., 2003) primers. The PCR protocol included an initial denaturation step at 98°C for 1 min, followed by 35 cycles of a denaturation step of 98°C for 30 sec, an annealing step of 68°C for 45 sec, and an elongation step at 72°C for 1 min, and a final elongation step at 72°C for 5 min. In a total reaction volume of 50 µl, the reaction included 1U Q5 High-Fidelity DNA Polymerase (New England Biolabs, USA), 1 x Q5 reaction buffer, 0.5 µM of each primer, 200 µM dNTP (New England Biolabs), 1 mg/mL BSA (Sigma-Aldrich, USA) and 1 µL of DNA. A negative control without DNA was included in each series of PCRs. The PCR products were purified using the NucleoSpin Gel and PCR Clean-up kit (Macherey-Nagel). The different primers used for the sequencing are 359F, 979F (5' CGATGCAACGCGAAGAAC 3') (Rajaniemi-Wacklin et al., 2005), 1092R (5' GCGCTCGTTGCGGGACTT 3') (Hrouzek et al., 2005), 1492R (5' TACGGYTACCTTACGACT 3') (Lane et al., 1990), and 23S30R. Sanger sequencing was performed at the GIGA-Genomics platform (University of Liège). The quality was checked and the sequences were manually trimmed in the program MEGA (version 7.0.26) (Kumar et al., 2016). For each strain, the five partial sequences were assembled using the program BioEdit (version 7.2.5) (Hall, 2011). The SSU rRNA and the ITS sequences were separated manually. Only the SSU rRNA sequences of the strains were used in this study.

References

Barbosa, M.; Berthold, D.E.; Lefler, F.W.; Laughinghouse IV, H.D. Diversity of the Genus *Brasilonema* (Nostocales, Cyanobacteria) in Plant Nurseries of Central Florida (USA) with the Description of Three New Species: *B. fioreae* sp. nov., *B. santannae* sp. nov. and *B. wernerae* sp. nov. *Fottea* **2021**, 21, 82-99, doi:10.5507/fot.2020.019.

Berthold, D.E.; Lefler, F.W.; Werner, V.R.; Laughinghouse IV, H.D. *Johannesbaptistia floridana* sp. nov. (Chroococcales, Cyanobacteria), a Novel Marine Cyanobacterium from Coastal South Florida (USA). *Fottea* **2020**, 20, 152-159, doi:10.5507/fot.2020.008.

Berthold, D.E.; Lefler, F.W.; Huang, I.-S.; Abdulla, H.A.N.; Zimba, P.; Laughinghouse IV, H.D. *Iningainema tapete* sp. nov. (Scytonemataceae, Cyanobacteria) from Greenhouses in Central Florida (USA) Produces two Types of Nodularin with Biosynthetic Potential for Microcystin-LR and Anabaenopeptins. *Harmful Algae* **2021a**, 101, 101969, doi:10.1016/j.hal.2020.101969.

Berthold, D.E.; Lefler, F.W.; Laughinghouse IV, H.D. Untangling Filamentous Marine Cyanobacterial Diversity from the Coast of South Florida with the Description of *Vermifilaceae* fam. nov. and Three New Genera: *Leptochromothrix* gen. nov., *Ophiophycus* gen. nov., and *Vermifilum* gen. nov. *Molecular Phylogenetics and Evolution* **2021b**, 107010, doi:10.1016/j.ympev.2020.107010.

Da Silva Malone, C.F.; Rigonato, J.; Laughinghouse, H.D.; Schmidt, É.C.; Bouzon, Z.L.; Wilmotte, A.; Fiore, M.F.; Sant'Anna, C.L. *Cephalothrix* gen. nov. (Cyanobacteria): Towards an Intraspecific Phylogenetic Evaluation by Multilocus Analyses. *International Journal of Systematic and Evolutionary Microbiology* **2015**, 65, 2993-3007, doi:10.1099/ijs.0.000369.

Ernst, A.; Becker, S.; Wollenzien, U. I.A; Postius, C. Ecosystem-Dependent Adaptive Radiations of Picocyanobacteria Inferred from 16S rRNA and ITS-1 Sequence Analysis. *Microbiology* **2003**, 149, 217-228, doi:10.1099/mic.0.25475-0.

Fernandez-Carazo, R.; Hodgson, D.A.; Convey, P.; Wilmotte, A. Low cyanobacterial diversity in biotopes of the Transantarctic Mountains and Shackleton Range (80-82°S), Antarctica. *FEMS Microbiology Ecology* **2011**, 77, 503-517, doi:10.1111/j.1574-6941.2011.01132.x.

Fernández-Carazo, R.; Namsaraev, Z.; Mano, M.J.; Ertz, D.; Wilmotte, A. Cyanobacterial Diversity for an Anthropogenic Impact Assessment in the Sør Rondane Mountains Area, Antarctica. *Antarctic Science* **2012**, 24, 229-242, doi:10.1017/S0954102011000824.

Hall, T. BioEdit: An Important Software for Molecular Biology. *GERF Bulletin of Biosciences* **2011**, 2, 60-61.

Hrouzek, P.; Ventura, S.; Lukešová, A.; Mugnai, M.; Mugnai, M.A.; Komárek, J. Diversity of Soil Nostoc Strains: Phylogenetic and Morphological Variability. *Algological*

Studies/Archiv für Hydrobiologie, Supplement Volumes No. 117 **2005**, 117, 251–264, doi: 10.1127/1864-1318/2005/0117-0251.

Jancusova, M.; Kovacik, L.; Pereira, A.B.; Dusinsky, R; Wilmotte, A. Polyphasic Characterization of 10 Selected Ecologically Relevant Filamentous Cyanobacterial Strains from the South Shetland Islands, Maritime Antarctica. *FEMS Microbial Ecology* **2016**, 92, fiw100, doi:10.1093/femsec/fiw100.

Kopf, M.; Möke, F.; Bauwe, H.; Hess, W.R.; Hagemann, M. Expression Profiling of the Bloom-Forming Cyanobacterium *Nodularia* CCY9414 under Light and Oxidative Stress Conditions. *ISME Journal* **2015**, 9, 2139-2152, doi:10.1038/ismej.2015.16.

Kumar, S.; Stecher, G.; Tamura, K. MEGA7: Molecular Evolutionary Genetics Analysis Version 7.0 for Bigger Datasets. *Molecular Biology and Evolution* **2016**, 33, 1870-1874, doi: 10.1093/molbev/msw054.

Lane, D.S. 16S and 23S rRNA Sequencing. In Nucleic Acid Techniques in Bacterial Systematics; Stackebrandt, E., Goodfellow, M., Ed; John Wiley and Sons: New York:, NY, USA, 1990; pp 115–148.

Lefler, F. W.; Berthold, D. E.; Laughinghouse, D.A. The Occurrence of *Affixifilum* gen. nov. and *Neolyngbya* (Oscillatoriaceae) in South Florida (USA), with the Description of *A. floridanum* sp. nov. and *N. biscaynensis* sp. nov. *Journal of Phycology* **2021**, 57, 92-110, doi:10.1111/jpy.13065.

Nadeau, T.L.; Milbrandt, E.C.; Castenholz, R.C. Evolutionary Relationships of Cultivated *Oscillatorians* (Cyanobacteria). *Journal of Phycology* **2001**, 37, 650-654, doi:10.1046/j.1529-817.2001.037004650.x.

Nübel, U.; Garcia-Pichel, F.; Muyzer, G. PCR Primers to Amplify 16S rRNA Genes from Cyanobacteria. *Applied Environmental Microbiology* **1997**, 63, 3327-3332.

Ohki, K.; Fujita, Y. Photoregulation of Phycobilisome Structure during Complementary Chromatic Adaptation in the Marine Cyanophyte *Phormidium* sp. C86. *Journal of Phycology* **2004**, 28, 803-808, doi:10.1111/j.0022-3646.1992.00803.x.

Rajaniemi-Wacklin, P.; Rantala, A.; Mugnai, M.A.; Turicchia, S.; Ventura, S.; Komarkova, J.; Lepisto, L.; Sivonen, K.; Komárková, J.; Lepistö, L.; Sivonen, K. Correspondence between Phylogeny and Morphology of *Snowella* spp. and *Woronichinia naegeliana*, Cyanobacteria Commonly Occurring in Lakes. *Journal of Phycology* **2006**, 42, 226-232, doi.org/10.1111/j.1529-8817.2006.00179.x.

Rippka, R.; Deruelles, J.; Waterbury, J.B.; Herdman, M.; Stanier, R.Y. Generic Assignments, Strain Histories and Properties of Pure Cultures of Cyanobacteria. *Microbiology* **1979**, 111, 1-61, doi:10.1099/00221287-111-1-1.

Sciuto, K.; Moro, I. Detection of the New Cosmopolitan Genus *Thermoleptolyngbya* (Cyanobacteria, Leptolyngbyaceae) Using the 16S rRNA Gene and 16S-23S ITS Region. *Molecular Phylogenetics and Evolution* **2016**, 105, 15-35, doi:10.1016/j.ympev.2016.08.010.

Sciuto, K.; Moschin, E.; Moro, I. Cryptic Cyanobacterial Diversity in the Giant Cave (Trieste, Italy): The New Genus *Timaviella* (Leptolyngbyaceae). *Cryptogamie, Algologie* **2017**, 38, 285-323. doi:10.7872/crya/v38.iss4.2017.285.

Soares, F.; Ramos, R.; Trovão, J.; Cardoso, S.M.; Tiago, I.; Portugal, A. *Parakomarekiella sesnandensis* gen. et sp. nov. (Nostocales, Cyanobacteria) Isolated from the Old Cathedral of Coimbra, Portugal (UNESCO World Heritage Site). *European Journal of Phycology* **2020**, 56, 301-315, doi:10.1080/09670262.2020.1817568.

Strunecký, O.; Komárek, J.; Johansen, J.; Lukešová, A.; Elster, J. Molecular and Morphological Criteria for Revision of the Genus *Microcoleus* (Oscillatoriales, Cyanobacteria). *Journal of Phycology* **2013**, 49, 1167-1180, doi:10.1111/jpy.12128.

Strunecky, O.; Raabova, L.; Bernardova, A.; Ivanova, A.P.; Semanova, A.; Crossley, J.; Kaftan, D. Diversity of Cyanobacteria at the Alaska North Slope with Description of Two New Genera: *Gibliniella* and *Shackletoniella*. *FEMS Microbiology Ecology* **2020**, 96, fiz189, doi:10.1093/femsec/fiz189.

Taton, A.; Wilmotte, A.; Šmarda, J.; Elster, J.; Komárek, J. *Plectolyngbya hodgsonii*: A Novel Filamentous Cyanobacterium from Antarctic Lakes. *Polar Biology* **2011,** 34, 181-191, doi:10.1007/s00300-010-0868-y.

Taton, A.; Grubisic, S.; Brambilla, E.; De Wit, R.; Annick Wilmotte. Cyanobacterial Diversity in Natural and Artificial Microbial Mats of Lake Fryxell (McMurdo Dry Valleys, Antarctica): a Morphological and Molecular Approach. *Applied Environmental Microbiology* **2003**, 69, 5157-5169, doi: 10.1128/AEM.69.9.5157-5169.2003.

Tavakoli, Y.; Mohammadipanah, F.; Te, S.H.; You, L.; Gin, K.Y.H. Biodiversity, Phylogeny and Toxin Production Profile of Cyanobacterial Strains Isolated from Lake Latyan in Iran. *Harmful Algae* **2021**, 102054, doi:10.1016/j.hal.2021.102054.

Wilmotte, A. Growth and Morphological Variability of Six Strains of *Phormidium* cf. *ectocarpi* Gomont (Cyanophyceae) Cultivated under Different Temperatures and Light Intensities. *Archiv für Hydrobiologie, Suppl. Algological Studies* **1988**, 80, 35-46.

Zarrouk, C. Contribution à l'Etude d'une Cyanobacterie: Influence de Divers Facteurs Physiques et Chimiques sur la Croissance et la Photosynthese de *Spirulina maxima* (Setchell et Gardner) Geitler. **1966**, Ph. D. Thesis. University of Paris, France.
