# Supplementary material for: ORPER: A Workflow for Constrained SSU rRNA Phylogenies
Source: Genes (Basel). 2021 Oct 29;12(11):1741. doi: 10.3390/genes12111741 (PMC8623055; doi:10.3390/genes12111741)
Supplement: Supplementary file 1 [file genes-12-01741-s001.zip › genes-1429420-Supp-files/Supplementary File S2.pdf]

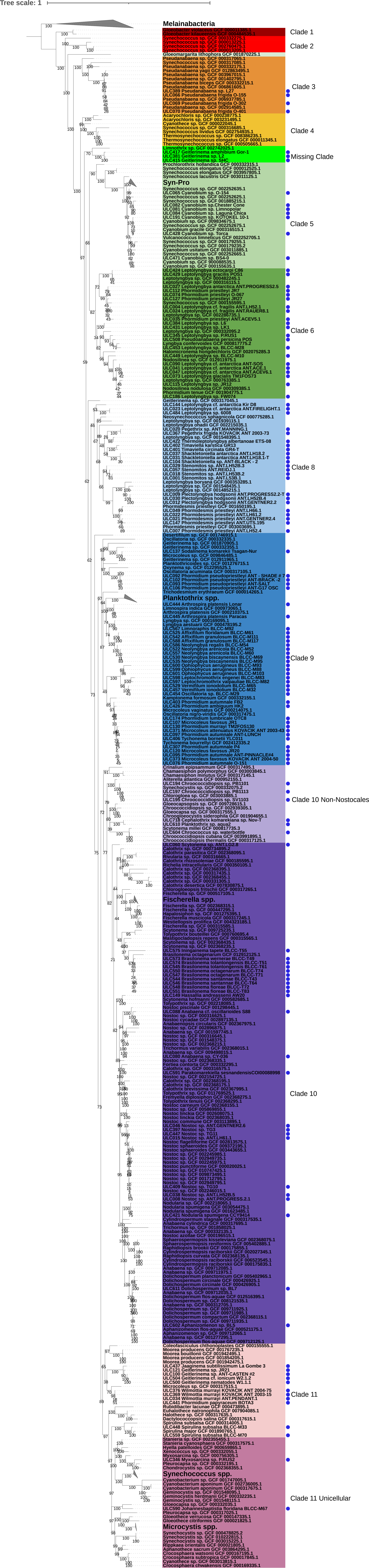
Tree scale: 1
Melainabacteria
Clade 1
Clade 2
Clade 3
Clade 4
Missing Clade
Syn-Pro
Clade 5
Clade 6
Clade 8
Planktothrix spp.
Clade 9
Clade 10 Non-Nostocales
Fischerella spp.
Clade 10
Clade 11
Synechococcus spp.
Clade 11 Unicellular
Microcystis spp.
